# Contrast-enhanced CT-based Radiomics for the Differentiation of Anaplastic or Poorly Differentiated Thyroid Carcinoma from Differentiated Thyroid Carcinoma: A Pilot Study

**DOI:** 10.1038/s41598-023-31212-8

**Published:** 2023-03-20

**Authors:** Jayoung Moon, Jeong Hoon Lee, Jin Roh, Da Hyun Lee, Eun Ju Ha

**Affiliations:** 1 Department of Radiology, Ajou University School of Medicine, Wonchon-dong, Yeongtong-gu, Suwon, 16499 Korea; 2 Department of Pathology, Ajou University School of Medicine, Wonchon-dong, Yeongtong-gu, Suwon, 16499 Korea

**Keywords:** Diseases, Endocrinology, Medical research, Oncology

## Abstract

Differential diagnosis of anaplastic thyroid carcinoma/poorly differentiated thyroid carcinoma (ATC/PDTC) from differentiated thyroid carcinoma (DTC) is crucial in patients with large thyroid malignancies. This study creates a predictive model using radiomics feature analysis to differentiate ATC/PDTC from DTC. We compared the clinicoradiological characteristics and radiomics features extracted from a volume of interest on contrast-enhanced computed tomography (CT) between the groups. Estimations of variable importance were performed via modeling using the random forest quantile classifier. The diagnostic performance of the model with radiomics features alone had the area under the receiver operating characteristic (AUROC) curve value of 0.883. The sensitivity, specificity, positive predictive value (PPV), negative predictive value (NPV), and accuracy were 81.7%, 93.3%, 97.7%, 64.5%, and 84.6%, respectively, for the differential diagnosis of ATC/PDTC and DTC. The model with both radiomics and clinicoradiological information showed the AUROC of 0.908, with sensitivity, specificity, PPV, NPV, and accuracy of 82.9%, 97.6%, 99.2%, 67.1%, and 86.5% respectively. Distant metastasis, moment, shape, age, and gray-level size zone matrix features were the most useful factors for differential diagnosis. Therefore, we concluded that a radiomics approach based on contrast-enhanced CT features can potentially differentiate ATC/PDTC from DTC in patients with large thyroid malignancies.

## Introduction

Anaplastic thyroid carcinoma/poorly differentiated thyroid carcinoma (ATC/PDTC) accounts for only 1–2%/2–15% of all thyroid malignancies, but over 50%/15% of deaths from thyroid cancer^1–3^. Despite modern therapies, ATC/PDTC remains a highly aggressive disease and is the main cause of death from thyroid cancer^1–3^. Since most ATC/PDTCs are considered to arise from the dedifferentiation of differentiated thyroid cancers (DTCs), i.e., the transformation of pre-existing papillary thyroid carcinomas (PTCs) or follicular thyroid carcinomas (FTCs) into more aggressive histologic types, radiologists should be familiar with the imaging features of ATC/PDTCs to avoid delayed diagnosis^1–3^.

Radiomics is a quantitative approach to medical imaging aimed at enhancing the data available to clinicians using advanced mathematical analysis^4,5^. Radiomics derived from the volumetric analysis of a whole tumor on contrast-enhanced computed tomography (CT) showed potential for early tumor detection, histologic grading, and predicting the recurrence of various head and neck cancers^5–8^. Radiomics has shown potential for predicting the BRAF^V600^^E^ mutation, extrathyroidal extension and recurrence, and cervical lymph node metastasis (LNM) in thyroid cancer^9–12^. However, no information exists on the potential of radiomics derived from CT to detect ATC/PDTC, i.e., to distinguish it from large DTCs, at an early stage. Given the histologic heterogeneity of tumor-harboring thyroid tissue and the dedifferentiation of DTC to ATC/PDTC during the pathogenic process, radiomics feature analysis may potentially reveal ATC/PDTC in patients with large DTCs^13,14^.

This retrospective study devised a radiomics approach based on CT features extracted from a volume of interest (VOI) to detect ATC/PDTC in patients with DTCs > 3.0 cm.

## Results

### Baseline clinicoradiological characteristics of patients with ATC/PDTC and DTC

The clinical and radiological characteristics of the patients with thyroid cancers included in this study are summarized in Table 1. The patients with ATC/PDTC (mean age, 60.9 ± 18.7 years: range: 14–85 years) were significantly older than those with DTC (mean age, 45.6 ± 15.7 years; range: 18–91 years) (p < 0.001). The sex distribution did not differ between the groups (p = 0.377). There were group differences in tumor size, number of lobes with tumor involvement, tumor margin, tumor growth pattern, intra-tumoral necrosis, calcification, organ invasion, and vascular invasion (all p < 0.05). Among them, the CT characteristics distinguishing ATC/PDTC from PTC were tumor size (mean size: 6.1 vs. 4.5 cm, p < 0.001), intra-tumoral necrosis (87.5% vs. 53.4%, p = 0.001), organ invasion (53.1% vs. 15.5%, p < 0.001), and vascular invasion (28.1% vs. 3.4%, p = 0.001). However, number of lobes with tumor involvement (p = 0.106), tumor margin (p = 0.379), tumor growth pattern (p = 0.123), and calcification (p = 0.257) did not differ between the two groups.

**Table 1:** Clinical and radiological characteristics of patients with thyroid cancers.

Variables	ATC/PDTC (n = 32)	DTC (n = 98)		*P-*value
		PTC (n = 58)	FTC (n = 40)	
Clinical characteristics				
Age (years, mean ± SD)	60.9 ± 18.7	44.2 ± 15.9	47.8 ± 15.5	< 0.001
≥ 55	21 (65.6)	14 (24.1)	27 (67.5)	
< 55	11 (34.4)	44 (75.9)	13 (32.5)	
Sex				0.377
Female	25 (78.1)	38 (65.5)	30 (75.0 )	
Male	7 (21.9)	20 (34.5)	10 (25.0)	
Radiologic characteristics				
Diameter (cm, mean ± SD)	6.1 ± 1.9	4.5 ± 1.4	4.7 ± 1.2	< 0.001
Location				0.092
Right lobe	12 (37.5)	30 (51.7)	15 (37.5)	
Left lobe	13 (40.6)	22 (37.9)	23 (57.5)	
Bilateral	7 (21.9)	5 (8.6)	1 (2.5)	
Isthmus	0 (0.0)	1 (1.7)	1 (2.5)	
Number of lobes with tumor involvement				0.017
1	25 (78.1)	53 (91.4)	39 (97.5)	
2	7 (21.9)	5 (8.6)	1 (2.5)	
Maximum tumor length (cm)				0.057
< 4.0	7 (21.9)	25 (43.1)	16 (40.0)	
≥ 4.0	25 (78.1)	33 (56.9)	24 (60.0)	
Tumor margin				0.005
Smooth	14 (43.8)	32 (55.2)	40 (100.0)	
Non-smooth	18 (56.3)	26 (44.8)	0 (0.0)	
Tumor growth pattern				< 0.001
Intra-thyroidal	12 (37.5)	33 (56.9)	40 (100.0)	
Extra-thyroidal	20 (62.5)	25 (43.1)	0 (0.0)	
Intra-tumor necrosis				0.002
Absent	4 (12.5)	27 (46.6)	14 (35.0)	
Present	28 (87.5)	31 (53.4)	26 (65.0)	
Calcification				0.008
Absent	9 (28.1)	24 (41.4)	32 (80.0)	
Present	23 (71.9)	34 (58.2)	8 (20.0)	
Organ invasion				< 0.001
None	15 (46.9)	49 (84.5)	40 (100.0)	
Esophagus/trachea/larynx	17 (53.1)	9 (15.5)	0 (0.0)	
Vascular invasion/encasement				< 0.001
None	23 (71.9)	56 (96.6)	40 (100.0)	
Carotid/jugular vein	9 (28.1)	2 (3.4)	0 (0.0)	
Lymphadenopathy				< 0.001
None	9 (28.1)	21 (36.2)	40 (100.0)	
Central (level VI)	5 (15.6)	13 (22.4)	0 (0.0)	
Ipsilateral (level II–V)	12 (37.5)	16 (27.6)	0 (0.0)	
Bilateral (level II–V)	6 (18.8)	8 (13.8)	0 (0.0)	
Retropharyngeal	7 (21.9)	0 (0.0)	0 (0.0)	
Mediastinal	8 (25.0)	1 (1.7)	0 (0.0)	
Nodal necrosis				< 0.001
Absent	15 (46.9)	51 (87.9)	40 (100.0)	
Present	17 (53.1)	7 (12.1)	0 (0.0)	
Nodal calcification				0.032
Absent	28 (87.5)	56 (96.6)	40 (100.0)	
Present	4 (12.5)	2 (3.4)	0 (0.0)	
Vascular invasion/encasement				0.001
Absent	26 (81.3)	57 (98.3)	40 (100.0)	
Present	6 (18.8)	1 (1.7)	0 (0.0)	
Distant metastasis at diagnosis				< 0.001
Absent	17 (53.1)	58 (100.0)	40 (100.0)	
Present	15 (46.9)	0 (0.0)	0 (0.0)	

Data in parentheses are percentages. ATC/PDTC, anaplastic thyroid carcinoma/poorly differentiated thyroid carcinoma; DTC, differentiated thyroid carcinoma; SD, standard deviation.

Regarding the lymph node characteristics, the rates of lymph node metastasis (LNM), nodal necrosis, nodal calcification, and vascular invasion/encasement were significantly higher in patients with ATC/PDTC (all p < 0.05). Among them, the CT characteristics distinguishing ATC/PDTC from PTC were nodal necrosis (53.1% vs. 12.1%, p < 0.001) and vascular invasion/encasement (18.8% vs. 1.7%, p = 0.007). However, cervical LNM (p = 0.603) and nodal calcification (p = 0.181) rates did not differ between the two groups. Distant metastasis at the initial diagnosis was only observed in patients with ATC/PDTC (46.9% vs. 0%, p < 0.001).

### Radiomics features extracted from CT scans of patients with ATC/PDTC and DTC

**Figure 1:**
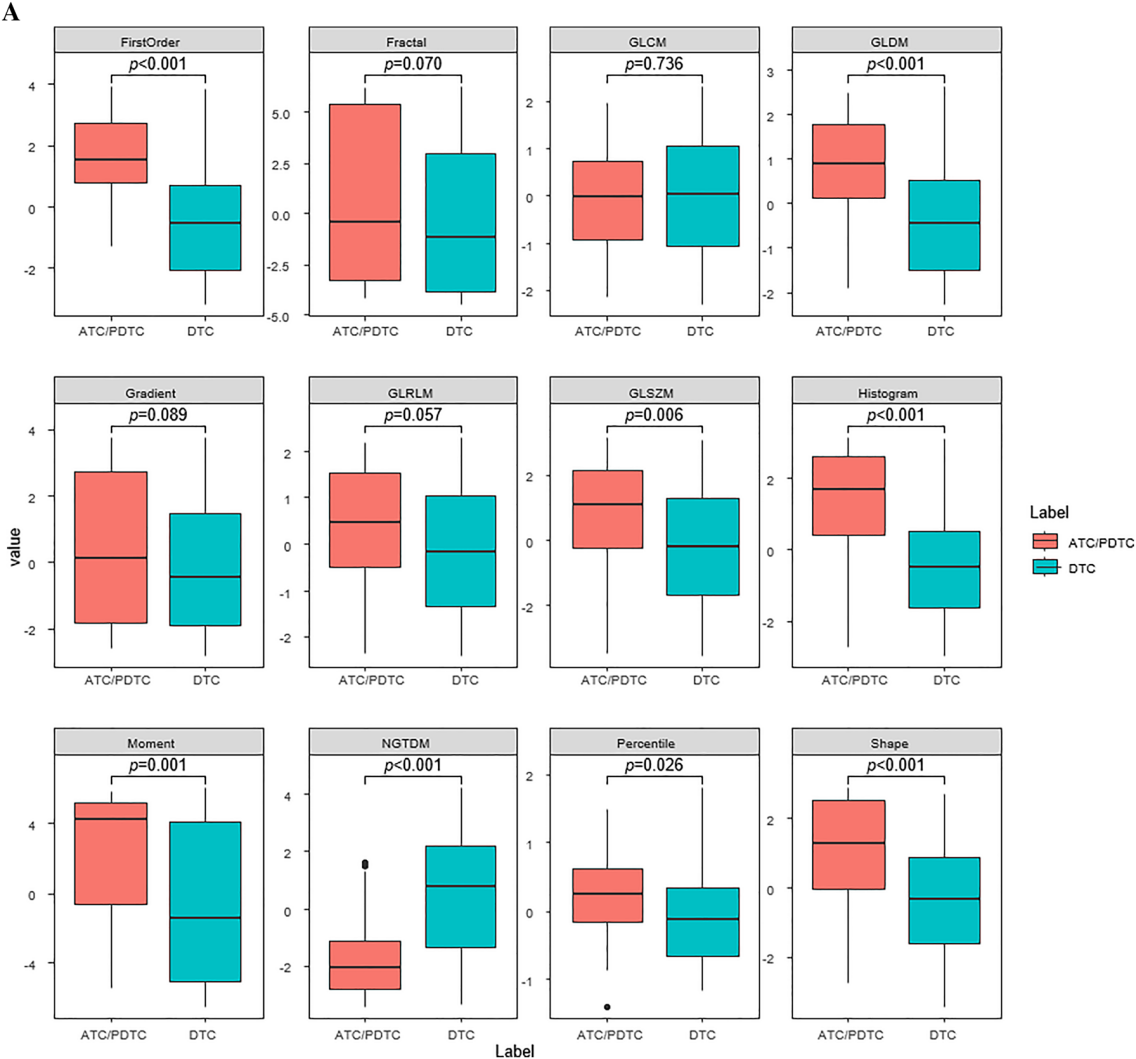

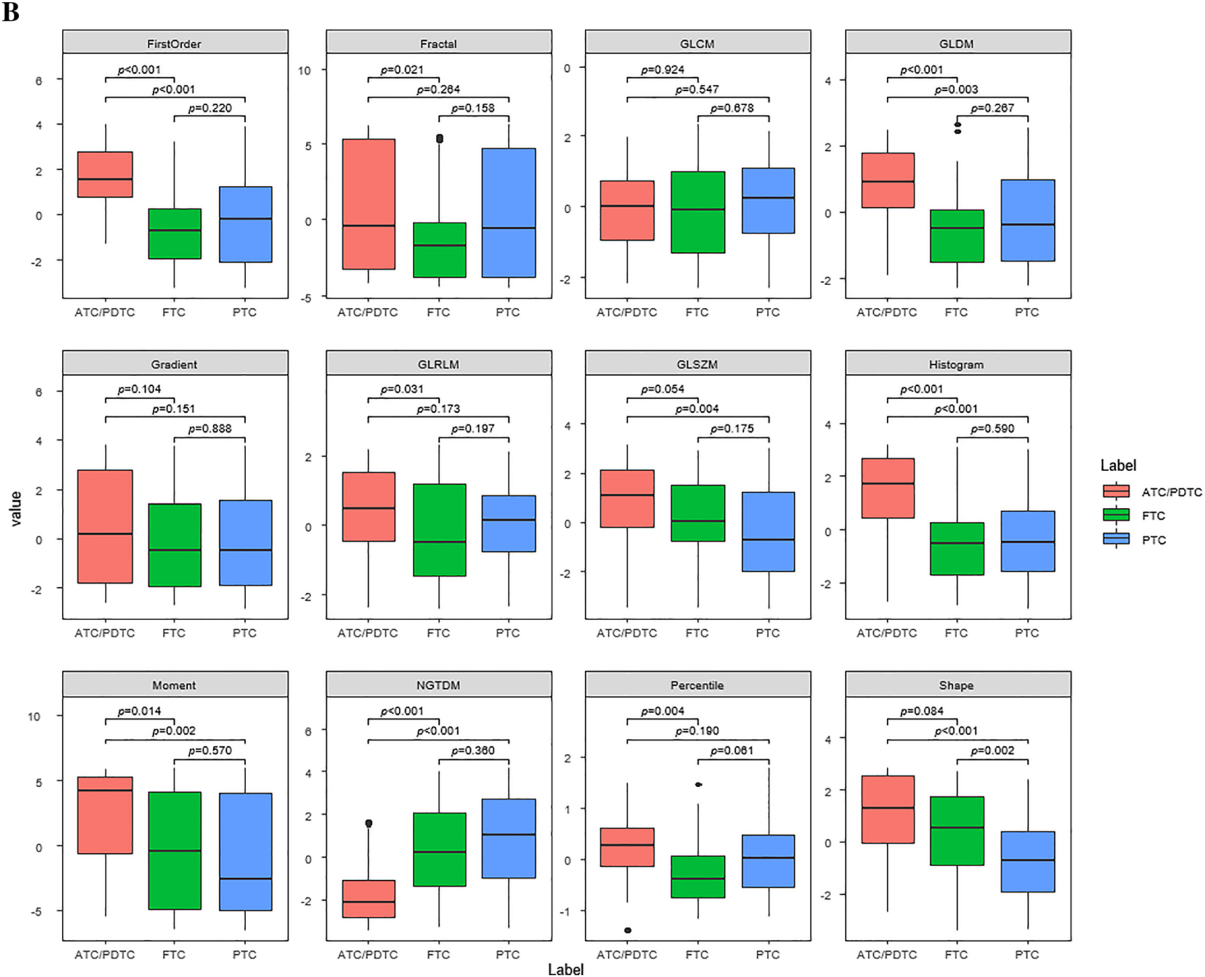
Box and whisker plots of the distribution of the representative values of the radiomics features on CT images of patients with ATC/PDTC and DTC (**A**), and patients with ATC/PDTC, FTC, and PTC (**B**).

Figure 1 shows representative values of radiomics features obtained through UMAP. Representative values of the first-order features, GLDM, GLSZM, histogram, moment, NGTDM, percentile, and shape features significantly differed between ATC/PDTC and DTC; the moment, GLSZM, shape, and first-order features differed most between the two groups.

**Figure 2:**
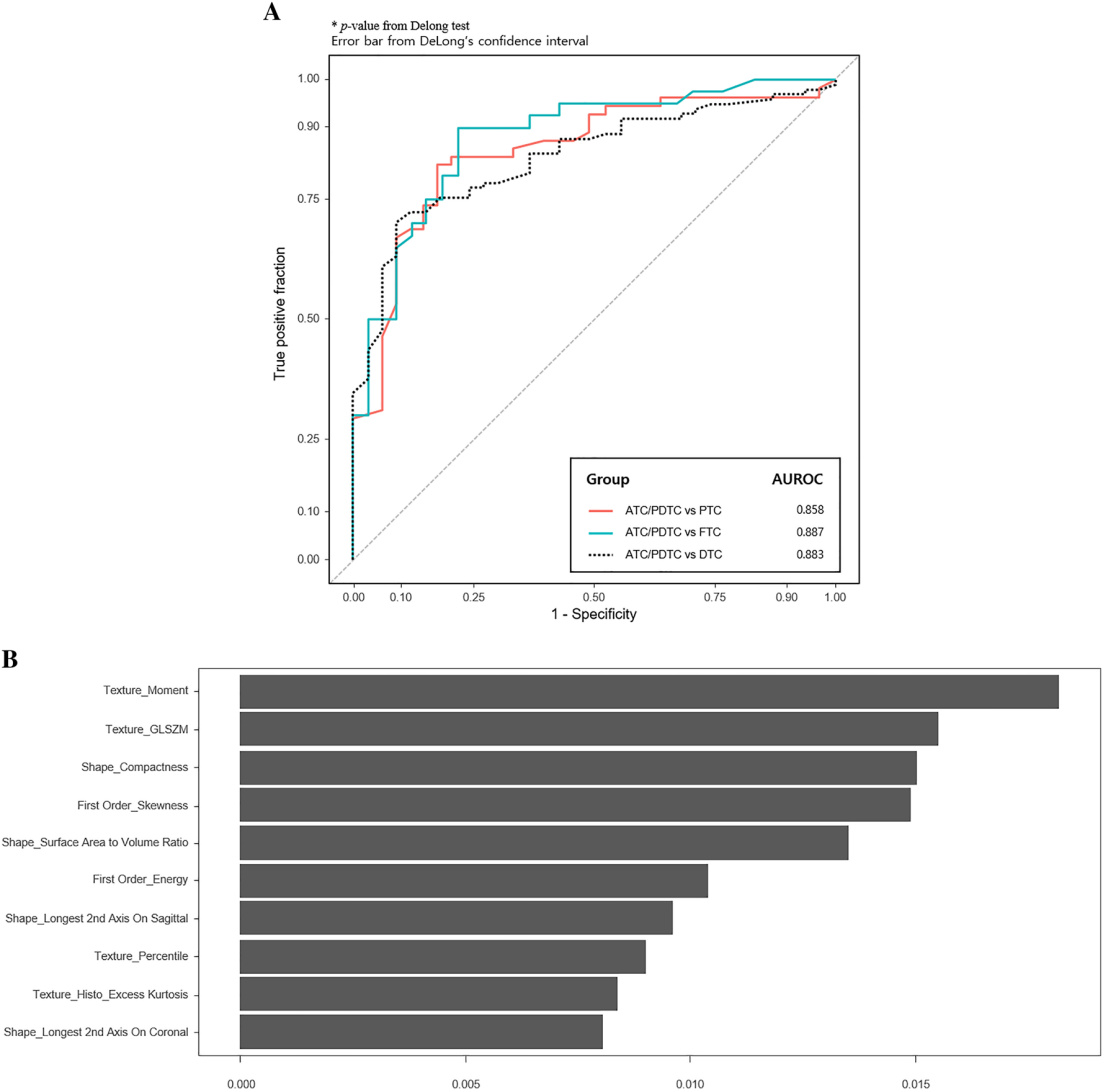
Receiver operating characteristic curves illustrating the diagnostic performance (for ATC/PDTC) of the models using radiomics alone (**A**) and the five most important features for distinguishing ATC/PDTC from DTC (**B**).

Table 2 and Fig. 2 show the diagnostic performance of the model using radiomics features extracted from CTs. The prediction model based on radiomic information alone had an area under the receiver operating characteristic (AUROC) curve of 0.883, with sensitivity, specificity, positive predictive value (PPV), negative predictive value (NPV), and accuracy of 81.7%, 93.3%, 97.7%, 64.5%, and 84.6%, respectively, for the differential diagnosis of ATC/PDTC and DTC. The AUROCs of the model were 0.858 and 0.887 for the differential diagnosis of ATC/PDTC with PTC and FTC, respectively.

**Table 2:** Ability of radiomics feature analysis to distinguish ATC/PDTC from DTCs > 3.0 cm based on contrast-enhanced CT.

Comparison	Sensitivity	Specificity	PPV	NPV	Accuracy
ATC/PDTC vs. DTC	81.7	93.3	97.7	64.5	84.6
ATC/PDTC vs. PTC	82.4	88.1	94.0	77.4	84.3
ATC/PDTC vs. FTC	85.0	90.5	92.8	84.6	87.4

Data are percentages. ATC/PDTC, anaplastic thyroid carcinoma/poorly differentiated thyroid carcinoma; DTC, differentiated thyroid carcinoma; FTC, follicular thyroid carcinoma; PTC, papillary thyroid carcinoma; PPV, positive predictive value; NPV, negative predictive value.

### The value of combined radiomics and clinicoradiological features to distinguish patients with ATC/PDTC and DTC

**Figure 3:**
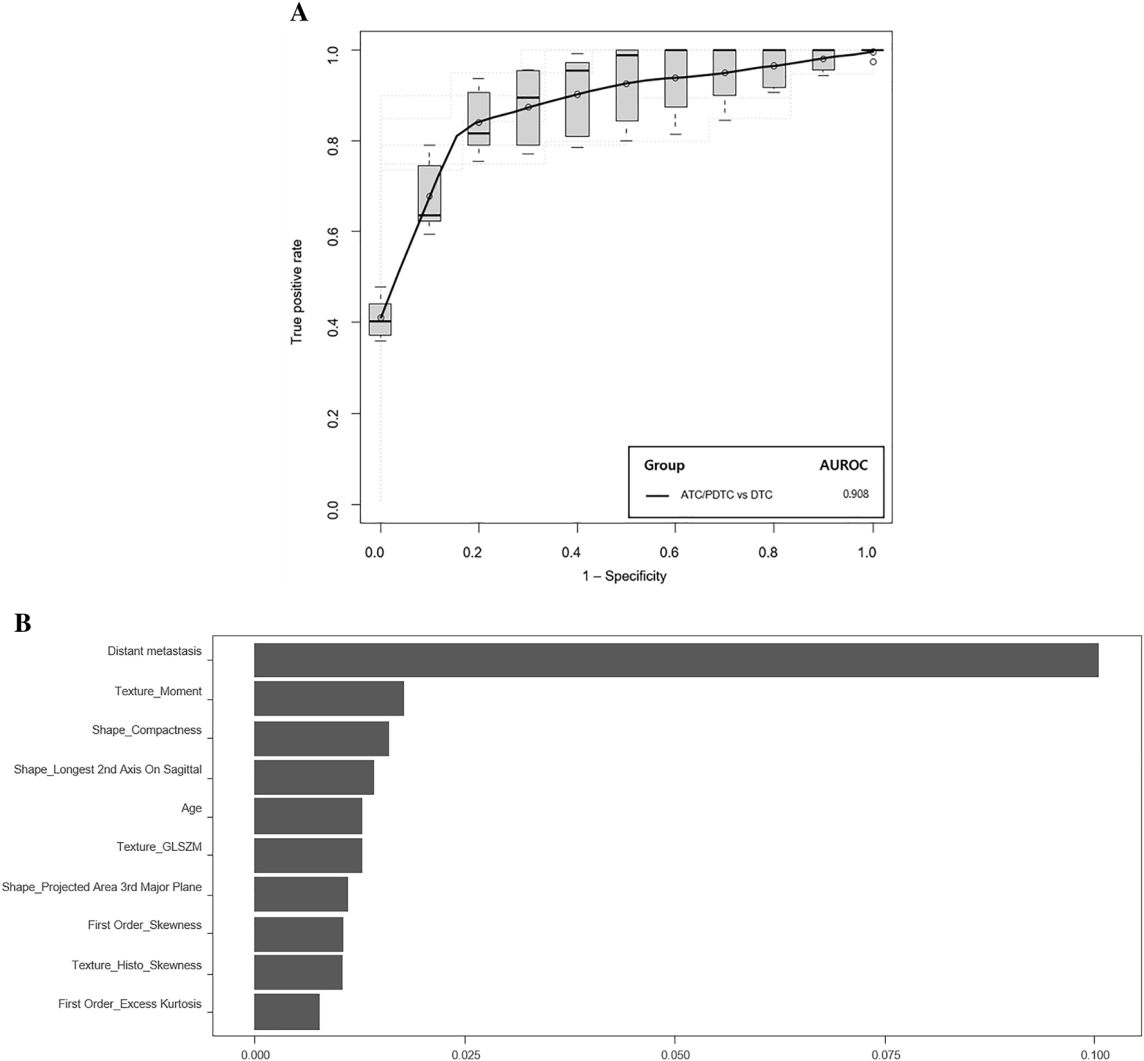
Receiver operating characteristic curves illustrating the diagnostic performance (for ATC/PDTC) of the models using combined features (**A**) and the five most important features for distinguishing ATC/PDTC from DTC (**B**).

Figure 3 shows the diagnostic performance of the prediction model based on combining radiomics and clinicoradiological information. The combined model showed an AUROC of 0.908, with sensitivity, specificity, PPV, NPV, and accuracy of 82.9%, 97.6%, 99.2%, 67.1%, and 86.5%, respectively, for the differential diagnosis of ATC/PDTC and DTC. There were no significant differences in the AUROCs between the two models. Distant metastasis, moment, shape, age, and GLSZM features differed most between ATC/PDTC and DTC.

## Discussion

This study demonstrated that a radiomics approach based on CT features extracted from a VOI could differentiate ATC/PDTC from DTC in patients with large thyroid malignancies, with an AUROC of 0.883. The model combining radiomics and clinicoradiological features had an AUROC of 0.908, with the most useful factors for differential diagnosis being distant metastasis, moment, shape, age, and GLSZM features. Image-based modeling of tumors with intratumoral heterogeneity combined with a conventional approach has potentials to overcome the clinical challenges of diagnosis and treatment planning.

The primary concern in large thyroid malignancies remains DTC, which represents > 90% of all thyroid cancers^15^. However, the differential diagnosis includes ATC/PDTC, which has a poor prognosis and may require a different treatment strategy^1–3^. Although sonography can evaluate a thyroid mass, patients with large neck masses frequently undergo contrast-enhanced CT for differential diagnosis^15^. As survival is improved by early multimodal treatments and increased use of head and neck imaging, radiologists must be familiar with the imaging features of ATC/PDTC to avoid delayed diagnosis^1–3,16^. ATC/PDTC usually presents as a large, heterogeneous, solid mass with increased vascularity, extensive necrosis, and local invasiveness^16–18^. However, ATC/PDTC sometimes shows nonspecific or overlapping imaging features with DTCs, especially for American Joint Committee on Cancer TNM system stage IVA and resectable stage IVB disease^19^. Accurate diagnosis of ATC/PDTC is possible with fine-needle aspiration in 50–80% of cases; however, false-negatives (atypical, benign, or non-diagnostic) and severity underestimation (i.e., cytological diagnosis of malignancy other than ATC/PDTC) are common, leading to poor clinical decision-making^20^. This study found that although conventional clinicoradiological features including older age, larger tumor size, intra-tumoral/nodal necrosis, and local invasiveness provided meaningful information for the differential diagnosis of ATC/PDTC and DTC, they overlapped, especially ATC/PDTC and PTC. Although nodal metastases occurred in 71.9% of ATC/PDTC patients at diagnosis, this was not statistically different from the rate among patients with PTC (63.8%). On the other hand, distant metastases were only present in 46.9% of ATC/PDTC patients upon presentation in this study, with the most common location being the lung.

Radiomics involves the extraction of quantitative metrics and radiomics features from medical images^4^. CT image-based radiomics feature analysis and modeling offer clinically feasible solutions to the challenges posed by heterogeneous tissue characteristics and shape, alone or in combination with demographic, histologic, or genomic data^5^. This study focused on the potential of radiomics approaches to differentiate ATC/PDTC from DTC based on the histologic heterogeneity of tumor-harboring thyroid tissue. Through application to CT imaging, radiomics was used to develop models integrating conventional clinicoradiological characteristics with quantitative radiomics features, to improve clinical decision-making and treatment planning of large thyroid malignancies. Our predictive model using radiomics features alone had an AUROC of 0.883 to detect ATC/PDTC, while the combined model had an AUROC of 0.908. The moment, GLSZM, and shape were the most useful radiomics features for differential diagnosis. The moment and GLSZM provide information on texture uniformity/heterogeneity^4,5^, and these values may serve as markers of intratumoral heterogeneity of the ATC/PDTC. The shape features provide information on the two- and three-dimensional properties of the tumor. The values reflect a more compact, sphere-like appearance of the ATC/PDTC than DTC^4,5^.

Although ATC/PDTC has a poor prognosis, in some cases survival can be improved by gross total resection combined with chemotherapy/targeted therapy and radiation therapy^1^. Accurate diagnosis is essential at the initial assessment; any unnecessary delay may lead to ATC/PDTC progressing from potentially resectable to unresectable and immediately threatening. The prognosis for ATC/PDTC remains poor with standard treatment, and the intratumoral heterogeneity of the tumor characteristics poses many clinical challenges in terms of diagnosis and treatment planning^13,21,22^. Advanced imaging techniques may help overcome these clinical challenges in thyroid oncology. Radiomics or radiogenomic profiling may improve outcomes by allowing for more personalized therapies.

This study had some limitations. First, we only included 32 patients with ATC/PDTC due to the low prevalence of this entity^1–3^. So far, imaging studies in ATC/PDTC patients have been extremely limited. To our knowledge, there have been only three studies on CT images of ATC/PDTC patients published since 2000, reporting 57, 32, and 10 cases, respectively^16–18^. Although the overall number of ATC/PDTC patients is small in this study, this is the first study to creates a predictive model using radiomics feature analysis to differentiate ATC/PDTC from DTC in patients with large thyroid malignancies. Further studies with larger populations and higher statistical power are needed to validate our results in a prospective design. Second, delineating cancer boundaries when drawing region of interests (ROIs) remains technically challenging. Defining an entire cancer volume on CT might be especially complicated for thyroid cancers because of image distortion caused by the contrast agent and the relatively low image resolution^6^. We delineated cancer boundaries based on consensus but did not evaluate interobserver agreement. Further studies using automated tumor segmentation and extraction of CT radiomics features are needed in the future. Third, feature repeatability and reproducibility investigations are currently limited to a small number of ATC/PDTC cancer populations. Fourth, we tried to differentiate ATC/PDTC only from DTC > 3.0 cm. The actual prevalence of thyroid cancers > 3.0 cm is relatively low in practice due to increased use of imaging studies. In addition, since the minimum size of ATC/PDTC over 10 years in our institution (included in this study) was 3.5 cm, we considered that it seems to be appropriate to compare with DTC patients over 3.0 cm for differential diagnosis. Fifth, we did not consider several key clinical factors such as tumor growth rate or symptoms to differentiate ATC/PDTC from DTC.

In conclusion, a radiomics approach based on contrast-enhanced CT features extracted from a VOI can potentially differentiate ATC/PDTC from DTC in patients with large thyroid malignancies.

## Materials and methods

### Study population

The institutional review board of our hospital reviewed and approved our retrospective study protocol. Written informed consent was waived because of the retrospective nature of the study. All experiments were performed in accordance with relevant guidelines and regulations. We reviewed the medical records of patients at our institution confirmed as ATC/PDTC between May 2008 and October 2021. We enrolled patients who met the following criteria: pathologically proven ATC/PDTC after thyroidectomy or biopsy at our institution; and a pretreatment neck contrast-enhanced CT examination. We identified 38 patients with ATC/PDTC. After excluding six patients who did not undergo pretreatment neck contrast-enhanced CT, or underwent a CT at another hospital or had a severe dental or motion artifact on CT, we finally enrolled 32 patients (mean age, 60.9 years; range: 14–85 years).

Control subjects with DTCs > 3.0 cm were enrolled during the same study period for comparison. We enrolled 98 patients with PTCs (n = 58, mean age, 44.2 years; range: 18–91 years) or FTCs (n = 40, mean age, 47.8 years; range: 18–82 years) meeting the following criteria: a pathologically proven PTC or FTC after thyroidectomy; tumor size > 3.0 cm; and a pretreatment neck contrast-enhanced CT examination performed at our hospital.

### Imaging techniques

All CT images were obtained using 64- to 128-channel multi-detector CT scanners (SOMATOM Definition Flash; Siemens Medical Solutions, Cary, NC, USA, or Brilliance; Philips Medical Systems, Best, Netherlands) with tube voltages of 100 and 120 kVp. Contrast-enhanced CT scanning was performed 40 s after initiation of an intravenous injection of a 90 mL bolus of iodinated nonionic contrast material (300–350 mgI/mL) into the right arm, with a subsequent injection of 20–30 mL normal saline for flushing at 3 mL/s using an automated injector. Finally, CT images were obtained with 0.5–0.6 mm collimation and reconstructed into axial images every 2.0 mm on a 512 × 512 matrix^15^.

### Imaging processing and analysis

Two radiologists (E.J.H. and D.H.L.) with experience in head and neck imaging (15 and 7 years, respectively) reviewed the CT images and achieved a consensus. The images were evaluated for the following: lobes with tumor involvement (one vs. two lobes); maximal tumor length (< 4.0 vs. ≥ 4.0 cm); tumor margins (smooth vs. non-smooth); tumor growth pattern (intrathyroidal vs. extrathyroidal growth); intra-tumoral necrosis (absent vs. present); calcifications (absent vs. present); organ invasion (esophagus, trachea, larynx, or recurrent laryngeal nerve); and vascular invasion/encasement (carotid artery encasement, internal jugular vein involvement). Tumor invasion of adjacent critical structures such as the trachea, esophagus, and common carotid artery was diagnosed if the tumor was in contact with at least one-half of the circumference of these organs, and if the plane between them was lost^16–18^. Tumor necrosis was considered present when the number of Hounsfield units (HU) was 10–30 and there was no significant difference in the number of HU (≤ 5 HU) between the pre-and post-contrast CT images, as determined by ROI analysis^23^. Central and lateral compartment lymphadenopathy was documented, along with retropharyngeal and mediastinal lymphadenopathy. Lateral and central compartment lymphadenopathy was further assessed for calcification, necrosis, and vascular invasion/encasement.

### Image processing and analysis

**Figure 4:**
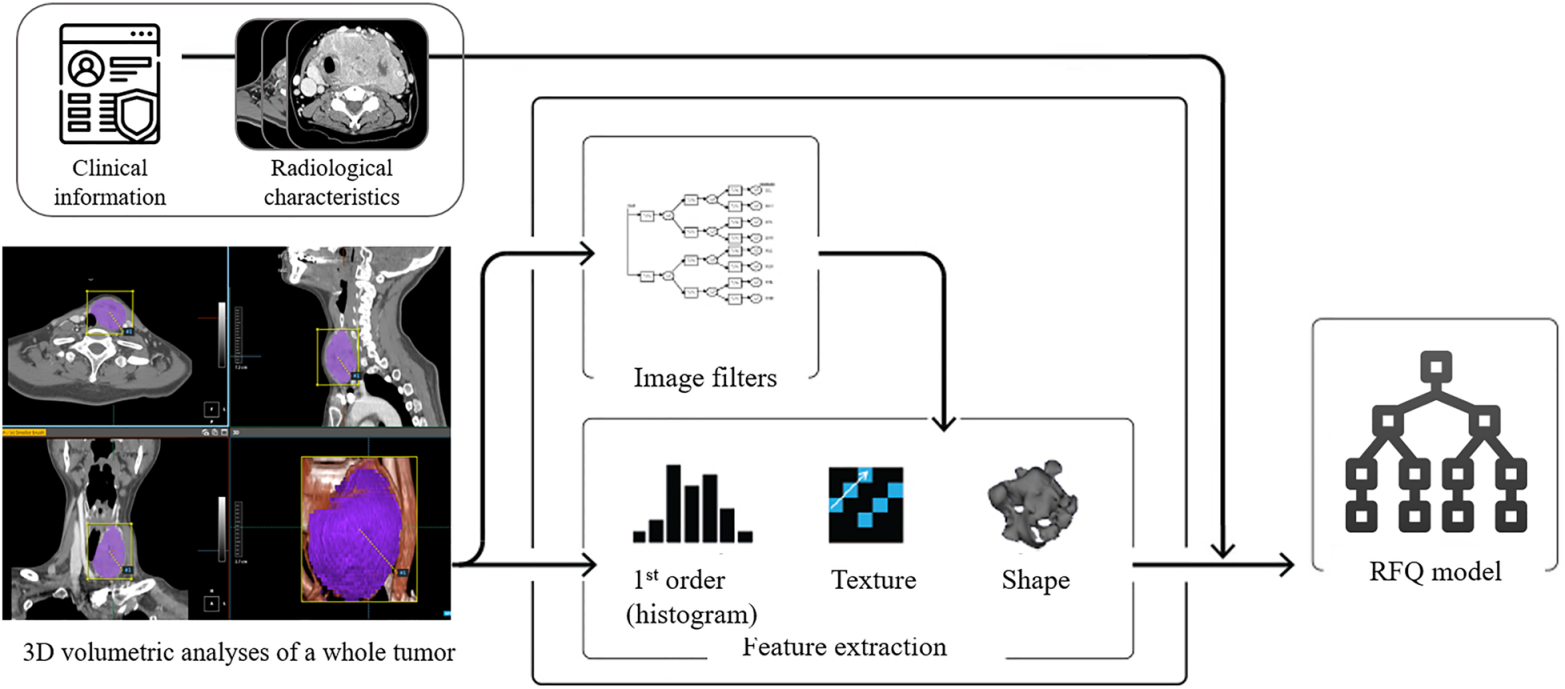
Workflow of the study.

Radiomics feature analysis was performed using PyRadiomics in A-VIEW software (Coreline Soft; Seoul, Korea; https://www.corelinesoft.com/aview-research-2)^24,25^. All patients’ CT scans were exported into the software, which semi-automatically created three-dimensional VOIs for each thyroid cancer. An experienced radiologist (E.J.H.) and two research scientists (E.K. and H.J.Y.) modified the VOIs on each axial image and achieved a consensus. For each VOI, 131 texture features were computed in 12 categories, including shape features (n = 26), first-order statistics features (n = 14), and histogram/percentile/gradient features (n = 17). Second-order statistical features were derived from the gray-level co-occurrence matrix (GLCM; n = 22), gray-level dependence matrix (GLDM; n = 14), gray-level run-length-matrix (GLRLM; n = 14), gray-level size zone matrix (GLSZM; n = 16), and neighborhood gray-tone difference matrix (NGTDM; n = 5). Higher-order statistics features included features from fractal analysis (n = 1) and moment features (n = 2). All features were transformed to the same scale through Z-score normalization. Figure 4 shows the workflow of this study.

### Statistical analysis

Statistical analyses were performed with R software (v. 4.1.2; R Foundation for Statistical Computing, Vienna, Austria). We used the Wilcoxon test to compare quantitative texture feature categories. Categorical clinical variables were compared using the Fisher’s exact test. Student’s t-test was employed to compare quantitative variables. To obtain representative values of the feature categories, we applied a non-linear dimension reduction algorithm, Uniform Manifold Approximation and Projection (UMAP), except in the case of the fractal analysis, which was composed of only one feature^26^. The discriminative power of the representative value of each feature category was evaluated based on geometric means of p-values obtained through group-wise comparisons.

The random forest quantile classifier (RFQ) is a machine learning model that solves class imbalance problems using a density-based approach^27^. We used an RFQ model to differentiate between ATC/PDTC and DTC. We used the Ishwaran-Kogalur importance method to quantify feature importance^28,29^. Area under the receiver operating characteristic (AUROC) curves were constructed from the radiomics features to determine the best predictive model and thresholds. The best predictive model was selected based on an AUROC derived via fivefold cross-validation. Since group-wise comparisons would result in class imbalance by data partitioning, we used a stratified fivefold cross-validation to keep samples for each class balanced as same as in the original percentage^30^. Hyperparameter tuning was performed with respect to Out-of-Bag error estimate to determine the optimal number of variables randomly sampled as candidates in each split and the size of node. The average AUROC derived via fivefold cross-validation was obtained from the arithmetic mean of the AUC^31^. The ROC curve of cross-validation was obtained by calculating the average value based on the true-positive rate and false positive rate for all of threshold points using cutpointr R package^32^. The threshold was determined by the highest Youden's J statistic. A p-value < 0.05 was considered statistically significant.

## Data Availability

The datasets used and/or analyzed during the current study available from the corresponding author on reasonable request.
